# Dietary Intake in the Lifelines Cohort Study: Baseline Results from the Flower Food Frequency Questionnaire among 59,982 Participants

**DOI:** 10.3390/nu14010048

**Published:** 2021-12-23

**Authors:** A. Mireille Baart, Elske M. Brouwer-Brolsma, Corine W. M. Perenboom, Jeanne H. M. de Vries, Edith J. M. Feskens

**Affiliations:** Division of Human Nutrition and Health, Wageningen University & Research, 6700 AA Wageningen, The Netherlands; elske.brouwer-brolsma@wur.nl (E.M.B.-B.); corine.perenboom@wur.nl (C.W.M.P.); jeanne.devries@wur.nl (J.H.M.d.V.); edith.feskens@wur.nl (E.J.M.F.)

**Keywords:** dietary intake, micronutrients, macronutrients, food groups, nutrition survey

## Abstract

The role of nutrition in health and disease is well established. However, more research on this topic is needed to fill gaps in our current knowledge. The Lifelines cohort study, a large Dutch prospective cohort study, was established as a resource for international researchers, aiming to obtain insight into the aetiology of healthy ageing. The study started with 167,729 participants, covering three generations, aiming to follow them for thirty years. This article describes the habitual dietary intake, assessed using the Flower Food Frequency Questionnaire (FFQ), among Lifelines cohort study participants at baseline, stratified by sex and different categories of age, socioeconomic status (SES) and body mass index (BMI). A total of 59,982 adults (23,703 men and 36,279 women), who completed the Flower FFQ and reported plausible habitual dietary intake, were included in the analyses. Median daily energy intake was higher in men (2368 kcal) than in women (1848 kcal), as well as macronutrient intake. Energy and macronutrient intake decreased with increasing age and BMI categories; no differences were observed between SES categories. Intake of most micronutrients was higher in men than in women. Differences were observed between age categories, but not between SES and BMI categories. Food groups were consumed in different amounts by men and women; differences between age, SES and BMI categories were observed as well. The Lifelines cohort study provides extensive dietary intake data, which are generalisable to the general Dutch population. As such, highly valuable dietary intake data are available to study associations between dietary intake and the development of chronic diseases and healthy aging.

## 1. Introduction

Nutrition plays an important role in health status, and an unhealthy diet is one of the key determinants of non-communicable morbidity and mortality [1]. For example, dietary patterns consisting of energy-dense, high-fat diets, with low fruit and vegetable intakes, are associated with an increased risk of developing diet-related non-communicable diseases such as cardiovascular diseases, type 2 diabetes and some cancers [2]. Although the existence of a relationship between nutrition and health status is evident, more research on this topic is warranted to fill the gaps in knowledge. For instance, only few well-established clear links between nutrition and cancer exist. Future research might show further important risk factors or protective factors, for example specific food components or broader dietary patterns such as plant-based diets [3]. Regarding type 2 diabetes, several associations between dietary factors and this disease have been reported; however, only few of these associations were graded as high quality of evidence. More well-conducted research, with more detailed assessment of diet, is needed to achieve high quality of evidence for these associations and to be able to give strong dietary recommendations [4]. Moreover, in a review article on diet and cardiovascular disease, it was concluded that future research is indispensable for furthering our understanding of the role of diet in this disease and for translating nutritional science into practice [5]. The above applies to many other diseases as well. Furthermore, many interactions with other factors, such as genetic background [6] or gut microbiome [7], have not yet been elucidated. Large epidemiological studies offer the opportunity to further investigate associations between nutrition and health status and interactions with other factors, and to disentangle underlying pathways [8].

The Lifelines cohort study, a multi-disciplinary prospective population-based cohort study in the north of The Netherlands, was established in 2006 as a resource for international researchers, aiming to obtain insight into the aetiology of healthy ageing [9]. It employs a broad range of investigative procedures in assessing the behavioural, socio-demographic, biomedical, physical and psychological factors which contribute to the health and disease of the general population, with a special focus on multi-morbidity and complex genetics. The study started with 167,729 participants, covering three generations, aiming to follow them for at least thirty years. Questionnaires on demographics, health and lifestyle, including dietary intake, are administered every eighteen months, and physical measurements as well as biological sampling are scheduled every five years. With this large body of data, the Lifelines database offers a unique opportunity to study complex interactions between environmental, phenotypic and genomic factors in the development of chronic diseases and healthy ageing, including diet−disease associations.

Within the Lifelines cohort study, data on habitual dietary intake is collected using the Flower Food Frequency Questionnaire (FFQ) [10]. This FFQ was especially developed for the Lifelines cohort study as an alternative to the regular comprehensive FFQ, which is a long and time-consuming questionnaire. The Flower FFQ consists of one main questionnaire and three short complementary questionnaires that are administered at different time points during a five-year period, reducing the time for filling out the questionnaire per occasion and with that participant burden. This is important as participants of the Lifelines cohort study must fill out many other questionnaires and undergo several physical measurements. Based on the literature, stable food consumption patterns over time can be assumed [11]. As such, a valid long-term estimate of the habitual dietary intake of participants is obtained, including data on the intake of energy, several macro- and micronutrients, and food items. Both the breadth of available variables and the large sample size of the Lifelines cohort provide the opportunity to perform well-powered stratified analyses to thoroughly investigate associations between dietary intake and the development of chronic diseases and healthy aging. The purpose of this article is to describe dietary intake among Lifelines cohort study participants at baseline. Data are presented separately for men and women and stratified by different categories of age, socioeconomic status (SES) and body mass index (BMI).

## 2. Methods

### 2.1. Study Population

By the end of 2006, recruitment of participants for the Lifelines cohort study started among inhabitants of the northern three provinces of The Netherlands (Friesland, Groningen and Drenthe). All general practitioner’s practices in this area that used computerised patient records (over 80% of practices) were requested to help with the recruitment. Within these practices, all patients in the age range of 25–50 years were invited by their general practitioner. Exclusion criteria included having a severe mental or physical illness, limited life expectancy (<5 years), and insufficient knowledge of the Dutch language to complete a Dutch questionnaire. Eligible participants received a first questionnaire and were invited to a Lifelines research facility for a comprehensive health assessment. During this visit, participants were also asked to indicate whether family members would be willing to participate in the study, and in case of a positive response, family members were invited as well. Children were only allowed to participate if one of their parents was included in the study. In addition to this recruitment strategy, inhabitants of the three northern provinces could also register themselves via the Lifelines website. In December 2013, the recruitment period was closed after reaching the target number of 165,000 participants. At that time, the total number of participants included was 167,729. A more detailed description of the total study population of the Lifelines cohort study can be found elsewhere [9].

The Lifelines cohort study is conducted according to the principles of the Declaration of Helsinki and in accordance with the research code of the University Medical Center Groningen (UMCG). The Lifelines cohort study is approved by the medical ethical committee of the UMCG, the Netherlands. All participants gave written informed consent.

### 2.2. Assessment of Dietary Intake

**Figure 1:**
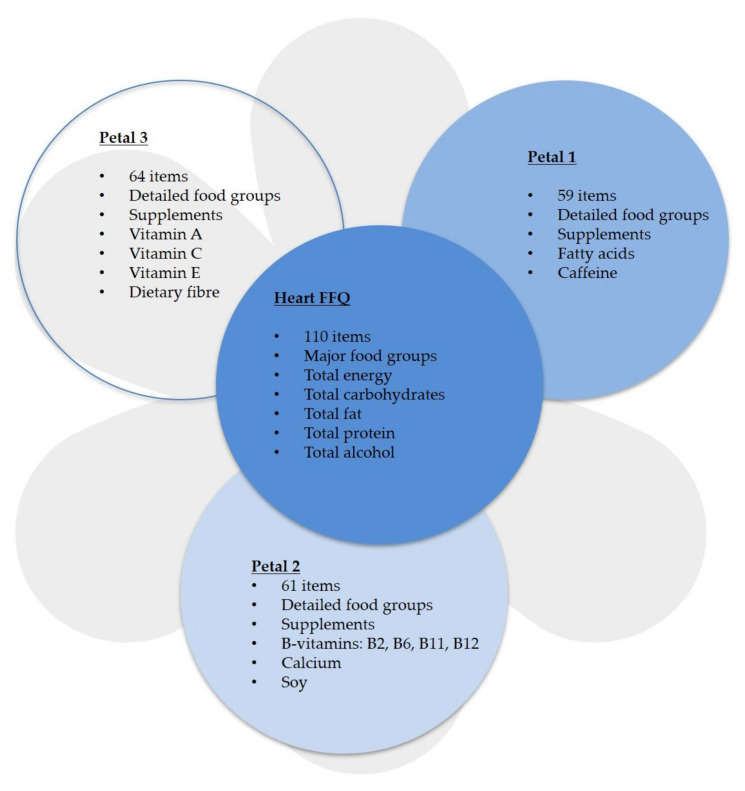
Flower FFQ. FFQ: Food Frequency Questionnaire.

Dietary intake at baseline was assessed using the validated Flower FFQ (Figure 1) [10]. The name Flower FFQ is derived from its design. The FFQ consists of one main questionnaire, which symbolises the heart of the flower, and three complementary questionnaires, which symbolise the flower petals. The heart FFQ contains 110 food items used to estimate intakes of major food groups, energy, carbohydrates, fat, protein and alcohol, but not in much detail. The three petal FFQs ask for detailed information on the types of food consumed within the food groups of the heart FFQ and also for supplement intake, to be able to estimate the intake of specific (micro)nutrients and food components. Thus, the difference between the heart FFQ and the petal FFQs is the degree of detail requested. For example, the heart FFQ provides basic information about the total amount of bread consumed, without information about the type of bread. More detailed information about bread type is provided by the third FFQ petal, in which the question “Did you eat bread?” is followed by questions on the type of bread (e.g., white, whole wheat). The first petal FFQ contains 59 food items used to estimate intakes of different types of fatty acids and caffeine; the second petal FFQ contains 61 food items used to estimate intakes of B-vitamins, calcium and soy; the third petal FFQ contains 64 food items used to estimate intakes of vitamin A, vitamin C, vitamin E and dietary fibre. Combined, the heart FFQ and the three petal FFQs cover 212 food items.

All adult participants of the Lifelines cohort study were invited to complete the Flower FFQ. The reference period of the heart and petal FFQs is one month, but it is assumed that food consumption patterns are stable over a longer period of time [11]. Therefore, the heart FFQ and the petal FFQs can be filled out at different moments during a study period. At the first assessment (between 2007 and 2013), participants received an invitation to fill out the heart FFQ. During three subsequent assessments (2011–2014, 2012–2015 and 2014–2017), they received an invitation to fill out one of the petal FFQs. The three petal FFQs were randomly distributed to adult participants during these subsequent assessments so that each participant received the petals in one out of six possible orders. Within the four different assessments, participants filled out the FFQs at time points that were fairly evenly distributed over the years and seasons. These four assessments are referred to as the baseline for dietary intake. At future assessments in the coming years, participants will be invited to complete the heart FFQ and the petal FFQs again, which will be referred to as follow-ups for dietary intake. Not all participants completed the four questionnaires. In this article, dietary intake from participants who completed the total Flower FFQ, i.e., the heart FFQ and all three petal FFQs, is described.

With combined data obtained from the heart FFQ and the three petal FFQs, the frequency of consumption of food items was assessed. Questions pertaining to frequency were completed by selecting answers ranging from “never” to “6–7 days per week”. Portion sizes were estimated using natural portions and commonly used household measures. In case a food item was reported in either only the heart FFQ or only in the petal FFQ, the food item was considered to be not consumed.

From data on food consumption obtained with the Flower FFQ, average daily intake of foods was calculated. The 212 food items were categorised into 30 food groups (Supplementary Table S1). Data on food consumption were also converted into daily energy and nutrient intake using data from the Dutch food composition database of 2011 [12].

To correct for potential under- or overreporting, we excluded participants with implausible habitual dietary intake, i.e., with energy intake <800 and >4200 kcal for men and <500 and >3500 kcal for women [13,14].

### 2.3. Assessment of Other Characteristics

Data on sex, age, ethnicity, SES, smoking and physical activity were obtained from questionnaires. Age was categorised into age groups used by the Health Council of the Netherlands for the recommendation of nutrients, as follows: 18–50 years, 51–70 years and >70 years [15]. SES was categorised based on education attainment, because education is more differentiating than income in the Dutch population [16], as follows: no education, primary education, lower vocational education, lower general secondary education (low); intermediate vocational education, higher general secondary education (moderate); higher vocational education and university education (high). Smoking was categorised as current, former and never smoker and included use of cigarettes, cigarillos, cigars and pipe tobacco. Physical activity was assessed with the short questionnaire to assess health-enhancing physical activity (SQUASH). The SQUASH has been shown to be substantially correlated with physical activity measured by accelerometry (correlation coefficient = 0.45) [17]. Using the SQUASH, the average number of minutes per week of various domains of physical activity (e.g., commuting, work, household chores, leisure time including, e.g., gardening and sports) were assessed. Based on Ainsworth’s compendium of physical activities [18], metabolic equivalent of task (MET) values were assigned to the specific physical activities. Subsequently, the total number of minutes per week of moderate to vigorous physical activity (MVPA) was calculated, using MET values of ≥4.0 to <6.5 for moderate physical activity and MET values ≥6.5 for vigorous physical activity.

Anthropometric measurements were conducted by well-trained staff at Lifelines research facilities. Height and body weight were measured without shoes and heavy clothing. Height was measured using the SECA 222 stadiometer (Seca GmbH, Hamburg, Germany); body weight was measured using the SECA 761 scale (Seca GmbH, Hamburg, Germany). BMI was calculated as kg/m^2^ and then categorised into normal weight (BMI < 25 kg/m^2^), overweight (25 ≤ BMI < 30 kg/m^2^) and obesity (BMI ≥ 30 kg/m^2^) [19].

### 2.4. Statistical Analyses

Data were first checked for normality using a Kolmogorov−Smirnov test and visual inspection of Q-Q normality plots. All continuous variables showed a skewed distribution and are therefore presented as medians with 25th–75th percentiles. Categorical variables are presented as numbers with percentages.

To explore potential selection bias, characteristics of participants who completed the total Flower FFQ and who did not complete the total Flower FFQ were compared first, using a Mann–Whitney U test for continuous variables and a Chi-square test for categorical variables, for men and women separately. Thereafter, analyses were performed among participants who completed the total Flower FFQ.

Daily energy and nutrient intake, as well as intake of food groups, were compared between men and women using a Mann–Whitney U test. Dietary intake was also compared between different categories of age, SES and BMI, using a Kruskal−Wallis test, for men and women separately. To identify which categories were different from each other, post hoc pairwise comparisons were performed, using a Bonferroni correction to adjust for multiple testing.

The results of all statistical tests were considered significant when the level of significance was lower than 5%, i.e., *p* < 0.05. Statistical analyses were performed with SPSS software (Version 25, IBM, Armonk, NY, USA).

## 3. Results

**Figure 2:**
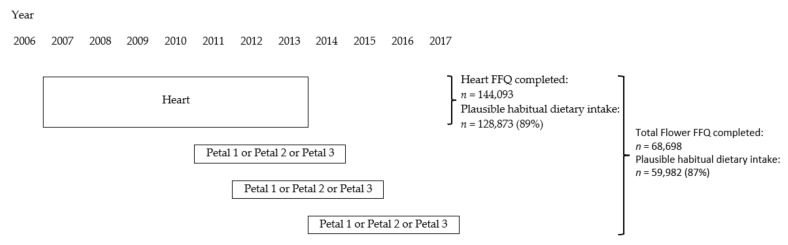
Participant flow and timeline of completing the Flower FFQ.

Figure 2 presents the participant flow. A total of 144,093 adults completed the heart FFQ. For 15,220 of these participants (11%), misreporting was highly likely because they reported unlikely low or high energy intake; 128,873 participants (89%) reported plausible habitual dietary intake. A total of 68,698 adults completed the total Flower FFQ. For 8716 of these participants (13%), misreporting was highly likely; 59,982 participants (87%) reported plausible habitual dietary intake. In this article, we primarily focus on the dietary intake data of these 59,982 participants (23,703 men and 36,279 women). Because of the large study population, almost all statistical tests were significant, even if differences were very small. Therefore, only striking and relevant differences are described.

Participants received an invitation to fill out the heart FFQ at the first assessment (between 2007 and 2013). During three subsequent assessments (2011–2014, 2012–2015 and 2014–2017), they received an invitation to fill out one of the petal FFQs. The three petal FFQs were randomly distributed to the participants during the follow-up so that each participant received the petals in one out of six possible orders.

### 3.1. Participant Characteristics

Table 1 presents characteristics of participants with plausible habitual dietary intake who completed the total Flower FFQ and who did not complete the total Flower FFQ. For participants who completed the total Flower FFQ, the median (25th–75th percentile) age was 47 (39–56) years for men and 46 (38–54) years for women. The majority of the participants were of white or east/west European ethnicity (98% for men, 97% for women). The distribution over the northern three provinces and over the three SES categories was approximately equal. The median (25th–75th percentile) MVPA was 285 (120–627) minutes per week for men and 245 (115–520) minutes per week for women, and the median (25th–75th percentile) BMI was 25.9 (23.9–28.2) kg/m^2^ for men and 24.8 (22.5–27.9) kg/m^2^ for women.

Compared to participants who did not complete the total Flower FFQ, the median age of participants who completed the total Flower FFQ was a little higher. Accordingly, for completers, the percentage of participants was lower in the 18–50 years age category and higher in the 51–70 years age category compared to non-completers. However, in both groups, most participants were classified in the 18–50 years age group. In general, completers had a higher SES compared to non-completers, and this difference was more pronounced in men than in women. Among completers, less current smokers but more former smokers were present than among non-completers, and completers were slightly more physically active than non-completers. The distribution of participants over the northern three provinces of the Netherlands, and the median BMI was comparable between the two groups. Data on ethnicity were missing for a large number of participants who did not complete the total Flower FFQ, and it is therefore hard to compare ethnicity between the two groups.

### 3.2. Energy and Nutrient Intake

Daily energy and nutrient intake are presented in Table 2. Median energy intake was higher in men (2368 kcal) than in women (1848 kcal), as well as absolute macronutrient and micronutrient intake, except for vitamin C, of which the intake was higher in women. In terms of energy percentage (En%), macronutrient intake in men and women were comparable, except for alcohol, of which the intake was 2.2 En% in men and 0.9 En% in women.

Table 3 presents daily energy and nutrient intake per age category. In general, median energy and macronutrient intake was highest in the lowest age category and decreased subsequently in the middle and highest age categories, both in men and women. A clear exception on the above concerns alcohol intake, which was highest in the middle age category, both in men and women. For micronutrient intake in general, no clear trend was observed with increasing age categories. In higher age categories, both in men and women, intake of retinol equivalents, vitamin C and calcium was a little higher compared to lower age categories, and in women only, this was true for folate, folate equivalents and vitamin B12 intake. On the other hand, intake of vitamin B6 and vitamin E was a little lower in higher age categories compared to lower age categories, particularly in men.

Table 4 presents daily energy and nutrient intake per SES category. No striking differences in daily energy and nutrient intake were observed among different categories of SES, except for alcohol intake in women, of which the intake was lowest in the low SES category and increased subsequently in the moderate and high SES categories.

Table 5 presents daily energy and nutrient intake per BMI category. A striking observation is that intake of energy and macronutrients was highest in participants with a normal weight and decreased with increasing BMI categories, both in men and women. An exception also concerns alcohol intake here, which was comparable in different BMI categories in men, whereas in women, alcohol intake was lower among obese participants compared to overweight participants and participants with a normal weight. Regarding micronutrient intake, no striking differences were observed among different categories of BMI.

**Table 2:** Daily energy and nutrient intake, obtained from the Flower FFQ (*n* = 59,982).

	Men (*n* = 23,703)		Women (*n* = 36,279)		
	Median	25th–75th Percentile	Median	25th–75th Percentile	
Energy (kcal)	2368	1974–2812	1848	1551–2179	***
Total carbohydrates (g)	255	209–308	203	167–242	***
(En%)	45.3	41.6–49.2	45.6	41.9–49.4	***
Mono- and disaccharides (g)	108	83–138	89	69–112	***
Polysaccharides (g)	144	117–175	112	91–133	***
Total fat (g)	95	76–120	73	58–91	***
(En%)	36.7	32.5–41.5	35.7	31.6–40.0	***
Saturated fatty acids (g)	33	26–41	26	20–32	***
Monounsaturated fatty acids (g)	34	26–42	25	20–32	***
Polyunsaturated fatty acids (g)	20	15–27	14	11–19	***
Eicosapentaenoic acid (EPA) (g)	0.04	0.01–0.09	0.04	0.01–0.09	***
Docosahexaenoic acid (DHA) (g)	0.06	0.02–0.13	0.06	0.02–0.12	***
Total protein (g)	84	71–98	71	60–81	***
(En%)	14.9	13.6–16.4	15.8	14.3–17.4	***
Vegetable protein (g)	37	30–44	29	24–35	***
Animal protein (g)	47	38–56	41	34–49	***
Alcohol (g)	6.8	2.6–15.3	2.6	0.4–6.9	***
(En%)	2.2	0.8–4.4	0.9	0.1–2.8	***
Fibre (g)	25	20–30	21	17–25	***
(En%)	2.0	1.7–2.3	2.2	1.9–2.5	***
Retinol equivalents (µg)	1146	868–1542	960	744–1232	***
Vitamin B2 (mg)	1.5	1.2–1.8	1.3	1.1–1.6	***
Vitamin B6 (mg)	1.5	1.3–1.8	1.3	1.1–1.5	***
Folate (present in food by nature) (µg)	253	210–303	229	190–272	***
Folate equivalents (µg)	261	214–317	234	193–282	***
Vitamin B12 (µg)	4.0	3.1–5.4	3.4	2.6–4.5	***
Vitamin C (mg)	92	66–123	96	69–127	***
Vitamin E (mg)	13	10–17	11	9–14	***
Calcium (mg)	986	786–1236	920	731–1134	***

* Significant difference between men and women. *** *p* < 0.001.

**Table 3:** Daily energy and macronutrient intake per age category, obtained from the Flower FFQ (*n* = 59,982).

	Men (*n* = 23,703)							Women (*n* = 36,279)						
	18–50 Years (*n* = 14,890)		51–70 Years (*n* = 8144)		>70 Years (*n* = 669)			18–50 Years (*n* = 24,361)		51–70 Years (*n* = 11,185)		>70 Years (*n* = 733)		
	Median	25th–75th Percentile	Median	25th–75th Percentile	Median	25th–75th Percentile		Median	25th–75th Percentile	Median	25th–75th Percentile	Median	25th–75th Percentile	
Energy (kcal)	2476	2071–2921	2215	1856–2633	2014	1704–2368	***abc	1892	1584–2222	1777	1494–2084	1679	1418–1956	***abc
Total carbohydrates (g)	271	224–324	232	192–279	213	178–251	***abc	210	174–249	190	157–226	183	153–212	***abc
(En%)	46.1	42.4–49.8	44.1	40.3–48.0	44.0	39.7–47.7	***ab	46.2	42.6–49.9	44.4	40.5–48.1	44.7	40.4–48.5	***ab
Mono- and disaccharides (g)	113	87–145	99	76–127	99	79–124	***ab	91	71–115	85	67–106	88	71–108	***ac
Polysaccharides (g)	154	127–185	131	108–158	111	93–133	***abc	116	96–138	103	85–123	93	76–109	***abc
Total fat (g)	99	79–123	89	71–113	82	64–105	***abc	75	59–93	69	55–86	65	51–81	***abc
(En%)	36.7	32.6–41.3	36.8	32.3–42.1	36.2	32.0–41.6	-	36.9	32.0–40.2	35.1	30.9–39.7	34.4	30.0–39.1	***abc
Saturated fatty acids (g)	34	27–42	31	24–39	29	23–37	***abc	26	21–33	25	20–31	24	19–31	***ab
Monounsaturated fatty acids (g)	35	28–44	31	24–40	28	22–36	***abc	26	21–33	24	19–30	21	17–27	***abc
Polyunsaturated fatty acids (g)	21	16–27	19	14–25	17	13–24	***abc	15	11–19	14	10–18	13	9–17	***abc
Eicosapentaenoic acid (EPA) (g)	0.04	0.01–0.08	0.06	0.02–0.10	0.05	0.03–0.09	***ab	0.03	0.01–0.08	0.06	0.02–0.10	0.05	0.01–0.09	***abc
Docosahexaenoic acid (DHA) (g)	0.05	0.02–0.11	0.08	0.03–0.15	0.08	0.04–0.13	***ab	0.05	0.01–0.11	0.08	0.03–0.15	0.05	0.01–0.09	***abc
Total protein (g)	86	73–100	81	69–94	74	64–86	***abc	71	60–81	71	60–82	65	57–77	***bc
(En%)	14.7	13.3–16.1	15.3	14.0–16.8	15.3	13.9–16.5	***ab	15.5	14.0–17.1	16.4	14.9–18.1	16.1	14.6–17.8	***abc
Vegetable protein (g)	38	31–46	34	28–41	29	24–36	***abc	29	24–35	28	23–33	25	21–30	***abc
Animal protein (g)	47	39–57	46	38–55	45	37–52	***abc	41	33–49	42	35–51	40	33–48	***ac
Alcohol (g)	6.6	2.5–13.3	8.5	2.8–16.8	6.3	1.6–12.5	***abc	2.5	0.3–6.7	3.3	0.7–9.3	1.4	0.0–6.4	***abc
(En%)	2	0.7–4.1	2.7	1.0–5.1	2.1	0.6–4.6	***ac	0.9	0.1–2.5	1.4	0.2–3.7	0.6	0.0–2.7	***abc
Fibre (g)	25	21–31	24	19–29	22	18–26	***abc	21	17–25	21	18–25	20	17–24	***abc
(En%)	2	1.7–2.3	2.1	1.8–2.4	2.1	1.8–2.4	***ab	2.1	1.8–2.4	2.3	2.0–2.6	2.3	2.0–2.6	***ab
Retinol equivalents (µg)	1126	853–1504	1182	894–1597	1181	902–1612	***ab	941	724–1197	1002	785–1301	995	788–1295	***ab
Vitamin B2 (mg)	1.5	1.2–1.8	1.5	1.2–1.8	1.4	1.2–1.6	***abc	1.3	1.0–1.5	1.3	1.1–1.6	1.3	1.1–1.6	***ab
Vitamin B6 (mg)	1.6	1.3–1.8	1.4	1.2–1.7	1.3	1.1–1.6	***abc	1.3	1.1–1.6	1.3	1.1–1.5	1.2	1.0–1.5	***abc
Folate (present in food by nature) (µg)	254	209–303	254	210–303	247	210–290	*abc	223	185–265	241	202–285	239	202–282	***ab
Folate equivalents (µg)	260	213–315	263	216–322	253	216–308	***a	227	188–273	247	206–299	246	206–302	***ab
Vitamin B12 (µg)	3.9	3.0–5.2	4.1	3.2–5.6	4.0	3.2–5.5	***a	3.3	2.5–4.3	3.6	2.8–4.9	3.5	2.7–4.6	***ab
Vitamin C (mg)	89	64–120	95	68–128	104	76–134	***abc	90	65–120	108	79–138	115	89–147	***abc
Vitamin E (mg)	14	11–18	13	10–17	12	9–16	***abc	11	9–14	11	9–14	10	10 8–13	***abc
Calcium (mg)	978	775–1231	1001	798–1247	1003	817–1211	***a	890	707–1100	974	788–1199	994	814–1196	***ab

* Significant difference between categories of age. * *p* < 0.05, *** *p* < 0.001. a: significant difference between 18–50 and 51–70 years, b: significant difference between 18–50 and >70 years, c: significant difference between 51–70 and >70 years.

**Table 4:** Daily energy and macronutrient intake per known SES category, obtained from the Flower FFQ (*n* = 58,971).

	Men (*n* = 23,296)							Women (*n* = 35,675)						
	Low SES (*n* = 6590)		Moderate SES (*n* = 8563)		High SES (*n* = 8143)			Low SES (*n* = 10,500)		Moderate SES (*n* = 14,314)		High SES (*n* = 10,861)		
	Median	25th–75th Percentile	Median	25th–75th Percentile	Median	25th–75th Percentile		Median	25th–75th Percentile	Median	25th–75th Percentile	Median	25th–75th Percentile	
Energy (kcal)	2355	1957–2834	2432	2013–2892	2319	1949–2725	***abc	1801	1512–2135	1868	1564–2199	1868	1576–2188	***ab
Total carbohydrates (g)	253	207–309	264	214–317	249	206–299	***abc	195	161–235	206	170–245	206	170–244	***ab
(En%)	45.1	41.2–49.0	45.5	41.8–49.3	45.4	41.8–49.1	***ab	45	41.1–48.9	45.8	42.1–49.5	45.9	42.4–49.6	***abc
Mono- and disaccharides (g)	109	82–141	111	85–143	104	81–132	***abc	87	68–111	90	71–114	88	70–110	***ac
Polysaccharides (g)	141	114–174	148	120–180	143	118–173	***ac	106	87–128	113	93–135	115	94–137	***abc
Total fat (g)	96	75–122	98	77–124	93	74–114	***abc	72	56–90	74	58–93	73	58–90	***abc
(En%)	36.9	32.3–42.2	36.9	32.5–41.8	36.5	32.6–40.8	***bc	35.6	31.2–40.3	35.9	31.9–40.3	35.5	31.7–39.5	***ac
Saturated fatty acids (g)	33	26–41	33	26–42	32	26–40	***abc	25	20–32	26	21–33	26	21–32	***ab
Monounsaturated fatty acids (g)	33	26–43	35	27–44	33	26–41	***abc	25	19–31	26	20–33	25	20–32	***abc
Polyunsaturated fatty acids (g)	21	15–28	21	15–27	19	14–25	***bc	15	11–19	15	11–19	14	11–18	***bc
Eicosapentaenoic acid (EPA) (g)	0.04	0.01–0.08	0.04	0.01–0.08	0.06	0.02–0.10	***bc	0.04	0.01–0.08	0.04	0.01–0.08	0.05	0.02–0.10	***bc
Docosahexaenoic acid (DHA) (g)	0.06	0.02–0.11	0.05	0.02–0.11	0.08	0.03–0.15	***bc	0.05	0.01–0.11	0.05	0.01–0.11	0.08	0.02–0.14	***bc
Total protein (g)	82	69–97	85	72–100	84	72–97	***abc	69	59–80	71	60–81	72	62–82	***abc
(En%)	14.6	13.3–16.1	14.7	13.4–16.2	15.3	14.0–16.7	***abc	15.8	14.2–17.5	15.6	14.1–17.3	16	14.5–17.6	***abc
Vegetable protein (g)	35	28–43	37	30–45	37	30–44	***ab	27	23–33	29	24–34	30	25–36	***abc
Animal protein (g)	46	38–56	47	39–57	47	38–55	***ac	41	33–49	41	34–49	41	33–49	*c
Alcohol (g)	6.7	2.5–15.4	6.7	2.5–15.0	6.8	2.7–15.5	**b	2	0.0–6.9	2.5	0.3–6.7	3.4	0.9–8.3	***abc
(En%)	2.2	0.7–4.4	2.1	0.8–4.3	2.4	0.9–4.5	***bc	0.8	0.0–2.8	0.9	0.1–2.6	1.3	0.4–3.2	***abc
Fibre (g)	24	19–29	25	20–30	25	21–30	***ab	20	17–24	21	17–25	22	18–26	***abc
(En%)	2	1.7–2.3	2	1.7–2.3	2.1	1.8–2.4	***bc	2.1	1.8–2.5	2.1	1.8–2.5	2.3	2.0–2.6	***abc
Retinol equivalents (µg)	1194	889–1633	1147	865–1544	1118	856–1465	***abc	961	737–1264	945	734–1207	980	764–1234	***ac
Vitamin B2 (mg)	1.5	1.2–1.8	1.5	1.2–1.8	1.5	1.2–1.8	***ac	1.3	1.1–1.6	1.3	1.0–1.6	1.3	1.1–1.6	-
Vitamin B6 (mg)	1.5	1.2–1.8	1.3	1.3–1.8	1.5	1.3–1.8	***abc	1.3	1.1–1.5	1.3	1.1–1.5	1.4	1.2–1.6	***abc
Folate (present in food by nature) (µg)	250	203–299	252	209–303	258	215–305	***abc	222	184–265	224	186–265	241	203–286	***bc
Folate equivalents (µg)	256	208–312	259	213–316	267	221–321	***abc	227	187–276	228	189–274	247	207–297	***bc
Vitamin B12 (µg)	4.0	3.0–5.4	4.0	3.0–5.3	4.1	3.1–5.5	***bc	3.4	2.6–4.5	3.3	2.6–4.4	3.5	2.7–4.7	***abc
Vitamin C (mg)	88	62–120	90	65–122	96	71–127	***abc	96	68–127	92	66–123	101	74–131	***abc
Vitamin E (mg)	13	10–17	14	11–18	13	10–17	***bc	11	9–14	11	9–14	11	9–14	**ac
Calcium (mg)	977	775–1232	987	785–1241	993	792–1233	-	925	735–1137	908	722–1121	929	743–1142	***ac

* Significant difference between categories of SES. * *p* < 0.05, ** *p* < 0.01, *** *p* < 0.001. a: significant difference between low and moderate SES, b: significant difference between low and high SES, c: significant difference between moderate and high SES.

**Table 5:** Daily energy and macronutrient intake per known BMI category, obtained from the Flower FFQ (*n* = 59,981).

	Men (*n* = 23,703)							Women (*n* = 36,278)						
	Normal Weight (*n* = 8875)		Overweight (*n* = 11,690)		Obesity (*n* = 3138)			Normal Weight (*n* = 18,608)		Overweight (*n* = 12,198)		Obesity (*n* = 5472)		
	Median	25th–75th Percentile	Median	25th–75th Percentile	Median	25th–75th Percentile		Median	25th–75th Percentile	Median	25th–75th Percentile	Median	25th–75th Percentile	
Energy (kcal)	2473	2081–2910	2324	1937–2765	2218	1823–2689	***abc	1898	1602–2219	1806	1519–2139	1766	1461–2100	***abc
Total carbohydrates (g)	272	226–326	249	204–300	232	188–283	***abc	210	175–248	196	161–234	192	155–233	***abc
(En%)	46.3	42.8–50.1	45	41.3–48.7	43.8	39.5–47.8	***abc	46.2	42.6–49.9	45.2	41.3–48.8	44.8	40.8–48.5	***bc
Mono- and disaccharides (g)	116	90–146	105	81–135	94	71–124	***abc	92	73–116	86	67–109	83	64–106	***abc
Polysaccharides (g)	153	126–184	141	114–170	134	108–165	***abc	115	95–137	108	88–130	107	86–130	***ab
Total fat (g)	98	79–122	94	74–118	92	71–116	***abc	75	59–93	71	57–90	70	55–88	***abc
(En%)	36.5	32.3–41.1	36.8	32.5–41.6	37	32.7–42.2	***ab	35.7	31.7–40.1	35.7	31.6–40.0	35.4	31.4–40.0	-
Saturated fatty acids (g)	34	27–42	32	26–40	31	24–40	***abc	27	21–33	25	20–32	25	19–31	***abc
Monounsaturated fatty acids (g)	35	28–43	33	26–42	32	25–42	***abc	26	20–33	25	20–31	25	19–31	***abc
Polyunsaturated fatty acids (g)	20	16–27	20	15–26	19	14–26	***abc	15	11–19	14	11–19	14	10–18	***abc
Eicosapentaenoic acid (EPA) (g)	0.04	0.01–0.09	0.05	0.02–0.09	0.05	0.02–0.09	***ab	0.04	0.01–0.09	0.04	0.01–0.09	0.04	0.01–0.08	***bc
Docosahexaenoic acid (DHA) (g)	0.06	0.02–0.12	0.06	0.02–0.13	0.07	0.02–0.13	***ab	0.06	0.02–0.012	0.06	0.02–0.13	0.06	0.02–0.11	***bc
Total protein (g)	86	73–99	83	71–97	82	69–97	***ab	71	60–82	70	60–81	70	59–81	*
(En%)	14.6	13.3–16.0	15	13.7–16.5	15.5	13.9–17.0	***abc	15.5	14.0–17.1	16	14.5–17.7	16.2	14.6–18.0	***abc
Vegetable protein (g)	39	32–47	36	29–43	34	27–42	***abc	30	25–35	28	23–34	27	22–33	***abc
Animal protein (g)	46	38–55	47	39–56	48	39–58	***abc	40	33–48	42	34–50	42	35–50	***ab
Alcohol (g)	6.7	2.6–13.3	6.9	2.6–15.9	6.7	1.9–15.8	***ac	2.9	0.7–7.1	2.6	0.3–7.1	1.3	0.0–5.2	***abc
(En%)	2.1	0.7–4.1	2.4	0.9–4.7	2.2	0.6–4.8	***abc	1.1	0.3–2.9	1	0.1–3.0	0.5	0.0–1.9	***abc
Fibre (g)	26	21–31	24	20–29	23	19–28	***abc	21	18–26	21	17–25	20	16–24	***abc
(En%)	2	1.7–2.3	2	1.7–2.3	2	1.7–2.3	***ab	2.2	1.9–2.5	2.2	1.9–2.5	2.2	1.9–2.5	-
Retinol equivalents (µg)	1151	879–1519	1142	865–1548	1155	844–1589	-	955	745–1221	963	745–1233	964	740–1273	*b
Vitamin B2 (mg)	1.5	1.2–1.8	1.5	1.2–1.8	1.4	1.2–1.8	***bc	1.3	1.0–1.6	1.3	1.1–1.6	1.3	1.0–1.6	*a
Vitamin B6 (mg)	1.5	1.3–1.8	1.5	1.2–1.8	1.4	1.2–1.7	***abc	1.3	1.1–1.5	1.3	1.1–1.5	1.3	1.1–1.5	***abc
Folate (present in food by nature) (µg)	261	217–313	250	207–298	243	200–293	***abc	231	192–275	227	189–270	223	183–265	***abc
Folate equivalents (µg)	269	222–327	258	212–312	248	204–303	***abc	236	195–285	233	193–280	227	86–273	***abc
Vitamin B12 (µg)	3.9	3.0–5.1	4.1	3.1–5.4	4.2	3.1–5.8	***abc	3.4	2.6–4.4	3.4	2.7–4.6	3.5	2.7–4.7	***ab
Vitamin C (mg)	93	67–125	91	66–122	88	63–120	***abc	96	70–127	97	70–127	94	66–124	***bc
Vitamin E (mg)	14	11–17	13	10–17	13	10–17	***abc	11	9–14	11	9–14	11	8–14	***ab
Calcium (mg)	998	797–1250	986	786–1232	957	746–1205	***bc	920	728–1135	924	739–1140	907	726–1120	**c

* Significant difference between categories of BMI. * *p* < 0.05, ** *p* < 0.01, *** *p* < 0.001. a: significant difference between normal weight and overweight, b: significant difference between normal weight and obesity, c: significant difference between overweight and obesity.

### 3.3. Food Intake

Daily food intake, categorised into food groups, is presented in Table 6. Intake of food groups was different for men and women. Striking differences were that men consumed more alcoholic beverages, bread, coffee, fat, oils and sauces, meat, potatoes, ready-made products, savoury snacks, soft drinks and sweets, whereas women consumed more fruits, tea, vegetables and water.

Table 7 presents daily food intake per age category. Both in men and women, intake of the following food groups decreased with increasing age categories: artificially sweetened beverages, bread, fat, oils and sauces, fruit juice, meat, pasta, ready-made products, rice, savoury snacks and soft drinks. On the other hand, intake of cheese, dairy, eggs, fruits, tea and vegetables increased with increasing age categories.

Table 8 presents daily food intake per SES category. Both in men and women, participants with a higher SES consumed more fruit juice, ready-made products, savoury snacks and vegetables, compared to participants with a lower SES. A difference observed in women only was that participants with a higher SES consumed more alcoholic beverages compared to participants with a lower SES.

Table 9 presents daily food intake per BMI category. A striking observation is that consumption of cake and cookies, fat, oils and sauces, savoury snacks, soft drinks and sweets was highest in participants with a normal weight and decreased with increasing BMI categories, whereas consumption of artificially sweetened beverages was lowest in participants with a normal weight and increased with increasing BMI categories, both in men and women.

**Table 6:** Daily food intake, categorised into food groups, obtained from the Flower FFQ (*n* = 59,982).

	Men (*n* = 23,703)		Women (*n* = 36,279)		
	Median	25th–75th Percentile	Median	25th–75th Percentile	
Alcoholic beverages (g)	107	36–215	29	4–85	***
Artificially sweetened beverages (g)	9	0–80	13	0–80	***
Bread (g)	163	120–208	116	83–148	***
Breakfast cereals (g)	0	0–6	0	0–8	***
Cake and cookies (g)	30	18–48	30	18–46	**
Cheese (g)	26	14–43	23	13–40	***
Coffee (g)	465	348–697	348	161–465	***
Dairy (g)	291	180–427	282	170–413	***
Eggs (g)	9	7–18	7	4–18	***
Fat, oils and sauces (g)	57	37–81	40	26–58	***
Fish (g)	13	4–22	12	3–20	***
Fruits (g)	105	44–203	133	68–205	***
Fruit juice (g)	21	0–96	21	0–54	***
Legumes (g)	7	0–16	4	0–11	***
Meat (g)	86	64–109	70	46–93	***
Nuts and seeds (g)	10	5–21	7	3–14	***
Pasta (g)	19	12–32	19	12–26	***
Potatoes (g)	95	53–138	71	40–104	***
Probiotics and drinks lowering cholesterol and blood pressure (g)	0	0–0	0	0–0	**
Ready-made products (g)	31	6–51	18	1–36	***
Rice (g)	20	8–32	16	6–25	***
Savoury snacks (g)	32	16–51	22	11–39	***
Soft drinks (g)	34	0–116	9	0–62	***
Soup (g)	36	22–89	36	22–72	***
Soy products (g)	0	0–0	0	0–0	***
Sweets (g)	34	17–57	25	12–42	***
Tea (g)	116	11–232	232	89–465	***
Vegan products other than soy (g)	0	0–0	0	0–0	***
Vegetables (g)	131	89–184	148	106–205	***
Water (g)	279	107–482	418	161–557	***

* Significant difference between men and women. ** *p* < 0.01, *** *p* < 0.001.

**Table 7:** Daily food intake, categorised into food groups, per age category, obtained from the Flower FFQ (*n* = 59,982).

	Men (*n* = 23,703)							Women (*n* = 36,279)						
	18–50 Years (*n* = 14,890)		51–70 Years (*n* = 8144)		>70 Years (*n* = 669)			18–50 Years (*n* = 24,361)		51–70 Years (*n* = 11,185)		>70 Years (*n* = 733)		
	Median	25th–75th Percentile	Median	25th–75th Percentile	Median	25th–75th Percentile		Median	25th–75th Percentile	Median	25th–75th Percentile	Median	25th–75th Percentile	
Alcoholic beverages (g)	107	36–223	110	38–217	64	17–139	***bc	27	4–72	36	7–101	14	0–64	***abc
Artificially sweetened beverages (g)	13	0–95	5	0–54	0	0–27	***abc	18	0–96	5	0–43	0	0–21	***abc
Bread (g)	169	129–214	149	111–198	132	100–166	***abc	119	84–151	114	82–145	108	78–137	***abc
Breakfast cereals (g)	0	0–6	0	0–6	0	0–6	***a	0	0–9	0	0–6	0	0–6	***ab
Cake and cookies (g)	30	17–47	30	18–48	35	23–50	***bc	30	18–46	30	17–47	35	23–50	***bc
Cheese (g)	24	12–41	29	17–46	30	18–47	***ab	21	11–37	28	17–43	29	18–42	***ab
Coffee (g)	465	322–697	465	348–697	465	348–523	***abc	348	45–465	465	241–581	348	232–465	***abc
Dairy (g)	290	177–430	292	183–421	307	204–422	-	276	164–400	293	183–422	309	236–440	***abc
Eggs (g)	9	7–18	14	7–18	18	7–18	***abc	7	4–18	9	7–18	18	7–18	***abc
Fat, oils and sauces (g)	59	40–84	53	34–77	47	30–70	***abc	42	27–61	36	23–53	33	21–49	***abc
Fish (g)	11	3–20	16	8–25	15	9–22	***ab	11	1–19	15	6–24	13	5–20	***abc
Fruits (g)	89	40–192	130	69–211	197	101–231	***abc	104	48–199	193	85–216	205	141–292	***abc
Fruit juice (g)	27	0–107	21	0–54	13	0–54	***ab	21	0–96	13	0–54	11	0–54	***ab
Legumes (g)	7	0–16	9	0–18	9	0–16	***ab	4	0–11	7	0–16	7	0–16	***ab
Meat (g)	90	66–112	80	61–103	71	48–93	***abc	72	49–95	67	41–89	62	36–75	***abc
Nuts and seeds (g)	11	5–21	10	4–21	6	3–14	***bc	7	3–14	7	3–15	4	1–10	***bc
Pasta (g)	24	13–39	19	8–26	12	3–18	***abc	19	12–32	13	8–19	8	3–13	***abc
Potatoes (g)	97	55–140	90	50–135	90	40–135	***ab	72	42–105	65	34–104	69	27–103	***ab
Probiotics and drinks lowering cholesterol and blood pressure (g)	0	0–0	0	0–0	0	0–0	-	0	0–0	0	0–0	0	0–0	*a
Ready-made products (g)	33	13–53	14	0–33	1	0–26	***abc	31	12–43	13	0–32	0	0–15	***abc
Rice (g)	20	10–34	16	6–26	10	0–20	***abc	16	6–26	15	4–24	10	0–16	***abc
Savoury snacks (g)	39	22–59	20	9–37	8	2–17	***abc	28	15–44	14	5–25	6	1–14	***abc
Soft drinks (g)	62	11–156	10	0–52	0	0–31	***abc	21	0–94	0	0–18	0	0–13	***ab
Soup (g)	36	22–72	36	22–89	36	22–89	-	36	22–45	36	22–72	36	22–72	***ab
Soy products (g)	0	0–0	0	0–0	0	0–0	***a	0	0–0	0	0–0	0	0–0	***ac
Sweets (g)	37	19–60	30	4–50	31	16–48	***ab	27	14–45	20	10–35	21	10–34	***ab
Tea (g)	89	11–232	116	18–241	232	116–465	***abc	232	89–465	232	116–465	348	161–465	***abc
Vegan products other than soy (g)	0	0–0	0	0–0	0	0–0	*	0	0–0	0	0–0	0	0–0	*c
Vegetables (g)	128	87–181	136	93–190	135	93–180	***a	143	100–199	162	118–217	152	109–200	***abc
Water (g)	279	107–557	279	107–418	279	139–418	***ab	386	139–579	418	193–557	418	161–557	***a

* Significant difference between categories of age. * *p* < 0.05, *** *p* < 0.001. a: significant difference between 18–50 and 51–70 years, b: significant difference between 18–50 and >70 years, c: significant difference between 51–70 and >70 years.

**Table 8:** Daily food intake, categorised into food groups, per known SES category, obtained from the Flower FFQ (*n* = 58.971).

	Men (*n* = 23,296)							Women (*n* = 35,675)						
	Low SES (*n* = 6590)		Moderate SES (*n* = 8563)		High SES (*n* = 8143)			Low SES (*n* = 10,500)		Moderate SES (*n* = 14,314)		High SES (*n* = 10,861)		
	Median	25th–75th Percentile	Median	25th–75th Percentile	Median	25th–75th Percentile		Median	25th–75th Percentile	Median	25th–75th Percentile	Median	25th–75th Percentile	
Alcoholic beverages (g)	107	33–229	107	36–227	107	39–208	-	21	0–76	27	4–71	27	4–71	***abc
Artificially sweetened beverages (g)	7	0–80	11	0–92	11	0–72	-	11	0–72	13	0–95	13	0–95	***ac
Bread (g)	165	123–213	166	124–211	153	113–201	***bc	118	85–148	117	83–149	117	83–149	-
Breakfast cereals (g)	0	0–3	0	0–6	0	0–10	***abc	0	0–5	0	0–6	0	0–6	***abc
Cake and cookies (g)	31	18–48	31	18–48	30	17–47	**bc	31	18–47	31	18–47	31	18–47	***bc
Cheese (g)	26	14–44	25	13–42	26	14–43	**ac	23	13–40	22	12–39	22	12–39	***ac
Coffee (g)	465	348–697	465	348–397	465	348–697	*b	465	232–581	348	116–465	348	116–465	***abc
Dairy (g)	288	177–423	294	185–431	291	178–426	***ac	288	178–418	284	172–417	284	172–417	***bc
Eggs (g)	14	7–18	9	7–18	9	7–18	***abc	7	4–18	7	4–18	7	4–18	***ab
Fat, oils and sauces (g)	58	38–84	59	39–84	53	35–76	***bc	39	25–58	41	27–59	41	27–59	***ac
Fish (g)	11	3–20	12	4–21	16	6–25	***bc	11	2–19	11	2–19	11	2–19	***bc
Fruits (g)	101	41–202	100	42–201	111	52–205	***bc	137	65–207	110	52–202	110	52–202	***abc
Fruit juice (g)	13	0–54	27	0–107	27	5–107	***abc	13	0–54	21	0–64	21	0–64	***abc
Legumes (g)	7	0–18	7	0–16	7	0–16	***ab	4	0–16	4	0–11	4	0–11	***abc
Meat (g)	86	64–110	89	66–111	83	62–105	***abc	70	46–92	72	50–95	72	50–95	***abc
Nuts and seeds (g)	9	3–19	10	4–21	12	5–22	***abc	6	2–14	7	3–14	7	3–14	***abc
Pasta (g)	19	11–32	19	13–32	20	13–45	***abc	13	8–20	19	12–26	19	12–26	***abc
Potatoes (g)	100	55–149	97	58–147	86	48–122	***bc	73	45–106	72	45–105	72	45–105	***bc
Probiotics and drinks lowering cholesterol and blood pressure (g)	0	0–0	0	0–0	0	0–0	***abc	0	0–0	0	0–0	0	0–0	***abc
Ready-made products (g)	14	0–36	32	12–52	33	12–52	***abc	13	0–32	26	6–36	26	6–36	***abc
Rice (g)	15	2–26	20	8–31	21	11–39	***abc	15	4–21	16	6–25	16	6–25	***abc
Savoury snacks (g)	26	12–46	34	19–54	33	17–52	***abc	18	8–33	25	13–42	25	13–42	***abc
Soft drinks (g)	35	0–126	42	3–136	26	0–94	***abc	0	0–47	13	0–90	13	0–90	***abc
Soup (g)	36	22–72	36	22–72	36	22–89	-	36	22–72	36	22–67	36	22–67	-
Soy products (g)	0	0–0	0	0–0	0	0–0	***abc	0	0–0	0	0–0	0	0–0	***abc
Sweets (g)	34	17–58	36	18–59	33	17–54	***abc	23	11–40	26	13–43	26	13–43	***abc
Tea (g)	89	0–232	89	11–232	134	36–348	***abc	232	80–348	232	89–465	232	89–465	***abc
Vegan products other than soy (g)	0	0–0	0	0–0	0	0–0	***abc	0	0–0	0	0–0	0	0–0	***abc
Vegetables (g)	122	81–171	126	86–178	146	102–200	***abc	137	96–188	143	101–197	143	101–197	***abc
Water (g)	279	107–557	279	107–557	279	107–418	-	418	193–697	418	161–579	418	161–579	***abc

* Significant difference between categories of SES. * *p* < 0.05, ** *p* < 0.01, *** *p* < 0.001. a: significant difference between low and moderate SES, b: significant difference between low and high SES, c: significant difference between moderate and high SES.

**Table 9:** Daily food intake, categorised into food groups, per known BMI category, obtained from the Flower FFQ (*n* = 59.981).

	Men (*n* = 23,703)							Women (*n* = 36,278)						
	Normal Weight (*n* = 8875)		Overweight (*n* = 11,690)		Obesity (*n* = 3138)			Normal Weight (*n* = 18,608)		Overweight (*n* = 12,198)		Obesity (*n* = 5472)		
	Median	25th–75th Percentile	Median	25th–75th Percentile	Median	25th–75th Percentile		Median	25th–75th Percentile	Median	25th–75th Percentile	Median	25th–75th Percentile	
Alcoholic beverages (g)	105	36–214	109	38–223	99	27–217	***abc	35	7–90	29	4–87	13	0–56	***abc
Artificially sweetened beverages (g)	0	0–54	13	0–92	29	0–143	***abc	7	0–54	13	0–93	29	0–139	***abc
Bread (g)	171	132–216	154	114–204	149	109–200	***abc	119	86–151	113	81–145	116	82–147	***ab
Breakfast cereals (g)	0	0–10	0	0–5	0	0–1	***abc	1	0–9	0	0–6	0	0–4	***abc
Cake and cookies (g)	32	19–50	30	17–47	27	15–43	***abc	31	18–47	30	17–46	29	16–44	***ab
Cheese (g)	25	13–42	26	14–43	26	14–43	**ab	23	13–40	23	13–40	22	13–39	*c
Coffee (g)	465	241–581	465	348–697	465	348–697	***ab	348	116–465	348	232–581	348	161–581	***ab
Dairy (g)	295	186–431	292	182–427	276	162–407	***bc	281	166–409	285	175–418	282	168–413	**a
Eggs (g)	9	7–18	14	7–18	14	7–18	***abc	7	4–18	7	4–18	9	4–18	***ab
Fat, oils and sauces (g)	59	40–84	56	37–80	53	34–77	***abc	42	27–60	39	25–57	37	24–54	***abc
Fish (g)	12	4–21	13	4–22	14	4–23	***ab	12	3–20	12	4–21	12	3–20	**c
Fruits (g)	106	46–203	105	45–204	97	39–201	***bc	130	69–205	137	70–206	115	47–204	***bc
Fruit juice (g)	27	0–107	21	0–96	21	0–96	***abc	21	0–96	21	0–54	13	0–54	***abc
Legumes (g)	7	0–16	7	0–16	7	0–16	-	4	0–11	4	0–11	4	0–11	***bc
Meat (g)	83	61–106	86	65–108	92	68–115	***abc	68	41–92	72	49–94	75	55–97	***abc
Nuts and seeds (g)	11	5–22	11	5–21	8	3–18	***abc	7	3–15	7	3–14	6	2–12	***abc
Pasta (g)	20	13–39	19	12–32	19	11–28	***abc	19	12–32	16	8–26	13	8–20	***abc
Potatoes (g)	97	55–143	95	53–136	90	47–132	***abc	71	40–104	72	40–104	68	39–104	-
Probiotics and drinks lowering cholesterol and blood pressure (g)	0	0–0	0	0–0	0	0–0	***abc	0	0–0	0	0–0	0	0–0	***ab
Ready-made products (g)	32	12–52	31	2–50	30	1–50	***ab	23	6–36	16	1–35	18	1–36	***ab
Rice (g)	20	9–34	17	6–31	16	4–27	***abc	16	8–26	15	5–24	15	4–24	***abc
Savoury snacks (g)	33	17–53	30	15–50	29	15–50	***ab	23	12–40	21	10–38	21	10–38	***ab
Soft drinks (g)	42	5–134	28	0–104	26	0–126)	***ab	13	0–67	5	0–52	0	0–57	***ab
Soup (g)	36	22–89	36	22–89	36	22–72	-	36	22–45	36	22–72	36	22–72	***ab
Soy products (g)	0	0–0	0	0–0	0	0–0	***abc	0	0–0	0	0–0	0	0–0	***abc
Sweets (g)	41	23–65	32	16–54	23	10–43	***abc	28	14–45	22	11–39	20	9–36	***abc
Tea (g)	116	18–322	116	11–232	80	0–232	***abc	241	116–465	232	89–465	232	80–465	***abc
Vegan products other than soy (g)	0	0–0	0	0–0	0	0–0	***abc	0	0–0	0	0–0	0	0–0	***abc
Vegetables (g)	134	93–189	129	87–181	129	86–180	***ab	148	106–205	149	106–205	147	102–203	*b
Water (g)	279	107–418	279	107–482	289	139–557	***abc	289	139–557	418	193–697	418	193–697	***abc

* Significant difference between categories of BMI. * *p* < 0.05, ** *p* < 0.01, *** *p* < 0.001. a: significant difference between normal weight and overweight, b: significant difference between normal weight and obesity, c: significant difference between overweight and obesity.

## 4. Discussion

In this article, we describe dietary intake among participants of the Lifelines cohort study at baseline. Median energy intake, as well as the intake of macronutrients, was higher in men than in women and decreased with increasing categories of age and BMI. No striking differences in energy and macronutrient intake were observed for different categories of SES. In regard to the intake levels stratified for age, SES and BMI, an exception to the above concerns alcohol intake. Alcohol intake was highest in the middle age category, not in the lowest age category, in both men and women. Among different SES and BMI categories, differences were observed in women: alcohol intake was lowest in the low SES category and highest in the lowest BMI category. Regarding micronutrients, the intake was higher in men than in women, except for vitamin C. Among different age categories, different intake of micronutrients was observed, but no clear trend was observed: for some micronutrients the intake was higher among higher age categories compared to lower age categories, whereas for other micronutrients the opposite was observed. No striking differences in micronutrient intake were observed among different categories of SES and BMI. Intake of most food groups differed between men and women, as well as between different categories of age, SES and BMI.

### 4.1. Generalisability

The study population comprised mainly inhabitants of the northern three provinces of the Netherlands. The population in the north of the Netherlands has a homogeneous composition and low migration rates relative to other parts of the Netherlands and is therefore highly suitable for a long-lasting follow-up study such as the Lifelines cohort study [9]. To obtain an impression of the generalisability of the data, we compared the results of the Lifelines population in the current article with results from the Dutch National Food Consumption Survey (DNFCS) [20], which is compiled from a representative sample (*n* = 2106; 1055 men, 1051 women) of the general Dutch population. Data on dietary intake in the DNFCS are presented by age categories, which are different from the age categories in the current article. Here, we describe data for the age category 18–50 years in the present population and for the category 31–50 years in the DNFCS, but comparisons are applicable to other age categories as well. Compared to the DNFCS, the present population had a slightly lower median intake of energy (2476 vs. 2647 kcal for men; 1892 vs. 1956 kcal for women). The intake of En% from carbohydrates and fat were slightly higher (for carbohydrates 46 vs. 43 En% for men and 46 vs. 45 En% for women; for fats 37 vs. 35 En% for men and 37 vs. 34 En% for women), whereas the intake of En% from protein was similar (15 En% for men and 16 En% for women), and the intake of En% from alcohol was lower (2.0 vs. 3.8 En% for men and 0.9 vs. 1.2 En% for women).

A comparison regarding intake of food groups between the present population and the DNFCS is more difficult to make, because within the DNFCS, food items were categorised into 17 food groups, and for the current article, food items were categorised into 30 food groups. Moreover, data on dietary intake in the DNFCS were collected using duplicate 24 h dietary recalls, which provide detailed information on dietary intake at two specific days, whereas in the Lifelines cohort study, dietary intake in the past month was assessed using an FFQ, which provides primarily information on food consumption patterns over time.

Because of this difference in dietary assessment method, we also compared our results to nutrient intake data obtained with a general FFQ, namely, data in the National Dietary Assessment Reference Database (NDARD) for the Dutch population (*n* = 1647; 857 men, 790 women), which was set up to serve as a reference database for new dietary assessment methods [21]. Data on energy and macronutrient intake in the NDARD are in the same range as in the present population and the DNFCS. It should be noted that NDARD participants lived in a relatively small part of the Netherlands around the city of Wageningen and had a higher SES compared to the general Dutch population, the DNFCS and Lifelines participants.

It is important to note that a majority of Lifeline participants who completed the heart FFQ at the first assessment did not complete all three petal FFQs at subsequent assessments.

Comparison of participants who completed the total Flower FFQ and who did not complete it showed that completers were a little older, had a higher SES, smoked less and were less physically active compared to non-completers. Participants who completed the total Flower FFQ may live healthier or may be more conscious about their health and are therefore more likely to complete all the questionnaires, which means that some selection bias occurred. However, in the present study population, a large variation in age, SES, smoking behaviour and physical activity still exists. Together with the high degree of comparability with data from the DNFCS and the NDARD, we conclude that data on dietary intake of the Lifelines population in the current article are generalisable to the general Dutch population.

### 4.2. Opportunities of Lifelines Data and Importance of Stratification

The large sample size of the Lifelines cohort and its heterogeneity in participant characteristics provide the opportunity to perform well-powered stratified analyses in studies on associations between dietary intake and the development of chronic diseases and healthy aging. To study these associations, stratification is important to control for confounding factors and effect modifiers, such as sex, age, SES and BMI [22,23]. In this article, we presented dietary intake for men and women separately, as well as for different categories of age, SES and BMI. Regarding energy and macronutrient intake, we observed a decrease in intake with both increasing age categories and increasing BMI categories, whereas no striking differences were observed between different categories of SES. The observation within different categories of BMI seems paradoxical; however, it is well known that a higher BMI is associated with misreporting, which could be explained by the tendency of participants to providing socially desirable answers [24]. Another explanation may be that participants with a higher BMI followed a calorie-restricted diet more often than participants with a lower BMI. In men, the percentages of participants that followed such a diet were 0.6, 2.0 and 5.0 for the normal weight, overweight and obese categories, respectively. In women, these respective percentages were 2.7, 7.5 and 11.5. Regarding intake of food groups, differences were observed between different categories of age, SES and BMI. This underlines the importance of stratification in research on dietary intake.

### 4.3. Strengths and Limitations

All self-reporting methods are prone to several types of error such as recall bias or the tendency to provide socially desirable answers [25]. An FFQ is not the best method to evaluate absolute nutrient intake and adequacy of nutrient intake. A specific limitation of an FFQ is that single foods are grouped into groups of food items, wherein the variation of reported intake may be underestimated. This results in a smaller distribution of nutrient intake, and consequently an underestimation of the prevalence rate of (in)adequate intake. Therefore, calculating and interpreting such prevalence rates should be done with caution. However, an FFQ is a reliable method to rank participants to their intake levels [26,27], which is also true for the specific Flower FFQ [10]. In epidemiologic studies on associations of dietary intake with diseases or health status, such as the Lifelines cohort study, ranking of participants according to their intake levels is usually more relevant than absolute levels of intake. Moreover, FFQs are the cheapest and most feasible method to assess food consumption patterns over a long time, which is another reason for their usefulness is epidemiologic studies. For the participants, however, an FFQ may be time-consuming and therefore considered burdensome to complete. This may result in the return of incomplete questionnaires and less valid answers at the end compared to the beginning of the questionnaire. As the Flower FFQ consists of four questionnaires that are administered at different time points, experienced burden and risk of bias may be lower for this FFQ than for a general FFQ. To illustrate, comparison of the time used to complete a regular FFQ and the Flower FFQ showed that completion of a regular FFQ took on average 43 min, and completion of the heart FFQ and the first, second and third petal took on average 24, 9, 8 and 9 min, respectively, adding up to a total of 50 min [10]. For the Lifelines cohort study, the different questionnaires were administered at different time points within a period of five years. Although stable food consumption patterns over time are assumed [11], changes in food consumption patterns may have occurred within these five years.

## 5. Conclusions

In conclusion, data on dietary intake obtained from the Flower FFQ among participants of the unique Lifelines cohort study are quite extensive and generalisable to the general Dutch population. As such, highly valuable dietary intake data are available in the Lifelines database to study associations between dietary intake and the development of chronic diseases and healthy aging.

## Figures and Tables

**Table 1:** Characteristics of participants with plausible habitual dietary who completed the total Flower FFQ (*n* = 59,982) and who did not complete the total Flower FFQ (*n* = 68,891).

	Men (*n* = 53,026)					Women (*n* = 75,847)				
	Completed Total Flower FFQ (*n* = 23,703)		Did Not Complete Total Flower FFQ (*n* = 29,323)			Completed Total Flower FFQ (*n* = 36,279)		Did Not Complete Total Flower FFQ (*n* = 39,568)		
	Median/n	25th–75th Percentile/%	Median/n	25th–75th Percentile/%		Median/n	25th–75th Percentile/%	Median/n	25th–75th Percentile/%	
Age (years)	47	39–56	44	34–51	*** ***	46	38–54	43	33–50	*** ***
Age category										
18–50 years	14,890	62.8	21,550	73.5		24,361	67.1	30,199	76.3	
51–70 years	8144	34.3	6738	23.0		11,185	30.8	8344	21.1	
>70 years	669	2.8	1035	3.5		733	2.0	1025	2.6	
Province					***					***
Friesland	8743	36.9	10,847	37.0		13,562	37.4	14,683	37.1	
Groningen	7461	31.5	8679	29.6		11,246	31.0	11,555	29.2	
Drenthe	6991	29.5	8820	30.1		10,641	29.3	11,978	30.3	
Other	502	2.1	725	2.5		812	2.2	1055	2.7	
Unknown	6	0.0	252	0.9		15	0.0	297	0.8	
Ethnicity					***					***
White, East/West European	23,232	98.0	20,286	69.2		35,167	96.9	28,212	71.3	
Other	308	1.3	448	1.5		628	1.7	789	2.0	
Unknown	163	0.7	8589	29.3		484	1.3	10,567	26.7	
SES					***					***
Low	6590	27.8	8515	29.0		10,500	28.9	11,475	29.0	
Moderate	8563	36.1	11,317	38.6		14,314	39.5	16,167	40.9	
High	8143	34.4	8781	29.9		10,861	29.9	11,047	27.9	
Unknown	407	1.7	710	2.4		604	1.7	879	2.2	
Smoking					***					***
Current smoker	4468	18.8	7435	25.4		5876	16.2	8718	22.0	
Former smoker	8722	36.8	9198	31.4		2480	34.4	11,737	29.7	
Never smoker	10,359	43.7	12,452	42.5		17,681	48.7	18,769	47.4	
Unknown	154	0.6	238	0.8		242	0.7	344	0.9	
Physical activity: MVPA (minutes per week)	285	120–627	280	90–630	** ***	245	115–520	240	90–480	*** ***
Physical activity category										
MVPA performance	19,562	82.5	23,179	79.0		31,361	86.4	32,707	82.7	
No MVPA performance	2246	9.5	3252	11.1		2743	7.6	4036	10.2	
Unknown or unreliable	1895	8.0	2892	9.9		2175	6.0	2825	7.1	
BMI (kg/m^2^)	25.9	23.9–28.2	26.0	23.9–28.5	** ***	24.8	22.5–27.9	25.0	22.5–28.4	*** ***
BMI category										
Normal weight	8875	37.4	10,868	37.1		18,608	51.3	19,595	49.5	
Overweight	11,690	49.3	13,909	47.4		12,198	33.6	13,048	33.0	
Obesity	3138	13.2	4546			5472	15.1	6925	17.5	
Unknown			15.5			1	0.0			

SES, socioeconomic status; MVPA, moderate to vigorous physical activity; BMI, body mass index. Continuous data are presented as median (25th–75th percentile), categorical data are presented as *n* (%). * Significant difference between participants who completed the total Flower FFQ and who did not complete the total Flower FFQ. ** *p* < 0.01, *** *p* < 0.001.

## Supplementary Materials

The following are available online at https://www.mdpi.com/article/10.3390/nu14010048/s1, Table S1: Classification of 212 food items into 30 food groups.
