# Supplementary material for: Dietary Intake in the Lifelines Cohort Study: Baseline Results from the Flower Food Frequency Questionnaire among 59,982 Participants
Source: Nutrients. 2021 Dec 23;14(1):48. doi: 10.3390/nu14010048 (PMC8746534; doi:10.3390/nu14010048)
Supplement: Supplementary file 1 [file nutrients-14-00048-s001.zip › nutrients-1467869-supplementary.pdf]

Supplementary table S1. Classification of 212 food items into 30 food groups.

Food group	Food item
Alcoholic beverages	Beer
	Red wine
	White wine
	Sherry/port/vermouth/madeira
	Spirits
	Other types of alcoholic beverages
	Non-alcoholic beer*
Artificially sweetened beverages	Light soft drinks/lemonade without sugar
Bread	Rusk/crispbread/crackers
	Brown bread
	Brown bread rolls
	Whole wheat bread
	Whole wheat bread rolls
	White bread
	White bread rolls
	Raisin/currant/muesli bread
	Raisin/currant/muesli bread rolls
	Other types of bread
	Other types of bread rolls
Breakfast cereals	Muesli/cruesli/cereals for porridge
Cake and cookies	Small biscuits
	Pastry/pie
	Gingerbread/cookie bars/nutritional biscuits
	Cake
	Filled cakes
	Other types of large cakes
Cheese	20+/30+ cheese/cheese spread
	40+ cheese/cheese spread
	48+ full-fat cheese/cheese spread
	Cream cheese/non-Dutch cheese
	Cheese with a hot meal
	Cheese in between meals
Coffee	Coffee
	Decaffeinated coffee
Dairy	Ready-to-drink breakfast
	Full-fat milk
	Semi-skimmed milk
	Skim milk
	Butter milk
	Chocolate milk
	Drinking yoghurt/dairy drink with flavor and sugar
	Other types of dairy drink
	Full-fat custard
	Full-fat plain yoghurt
	Low-fat plain yoghurt
	Low-fat (fruit) yoghurt
	Curd cheese/fruit curd
	Ready-made porridge
	Ice cream/milk based ice cream
	Other types of custard/yoghurt/curd
	Whipped cream
	Semi-skimmed/low-fat coffee creamer
	Full-fat coffee creamer/coffee cream

	Full-fat/semi-skimmed diet coffee creamer<br>Coffee creamer<br>Regular milk (in coffee)
	Other types of coffee creamer
Eggs	Boiled egg<br>Fried egg
Fat, oils and sauces	Gravy<br>Mayonnaise<br>Halvanaise/french fries sauce/non-red sauces<br>Dressing/salad dressing<br>Clear dressing without oil<br>Mayonnaise (snacks)<br>Halvanaise/french fries sauce/non-red sauces (snacks)<br>Cream with a hot meal<br>Satay sauce with a hot meal<br>Other types of hot sauces with a hot meal<br>Butter (on bread)<br>Semi-skimmed butter (on bread)<br>Margarine tub (on bread)<br>Margarine packet (on bread)<br>Diet margarine (on bread)<br>Margarine plant sterols (on bread)<br>Low-fat margarine (on bread)<br>Diet low-fat margarine (on bread)<br>Low-fat margarine plant sterols (on bread)<br>Other types of spread (on bread)<br>Butter (preparation)<br>Margarine tub (preparation)<br>Margarine packet (preparation)<br>Diet margarine (preparation)<br>Margarine plant sterols (preparation)<br>Liquid margarine (preparation)<br>Low-fat margarine plant sterols (bread)<br>Solid baking and roasting product (preparation)<br>Liquid baking and roasting product (preparation)<br>Olive oil/peanut oil (preparation)<br>Sunflower oil/other types of oil (preparation)<br>Lard/bacon fat<br>Unknown type of baking and roasting product
Fish	Salad with fish (on bread)<br>Salad with fish (on toast)<br>Fish on baguette or toast<br>Fish sticks<br>Gourmet/kibbeling<br>Lean fish<br>Fatty fish<br>Herring<br>Trout/tuna<br>Other types of fish<br>Shellfish
Fruits	Apple sauce<br>Citrus fruits<br>Apple/pear/banana<br>Strawberry<br>Kiwi<br>Other types of fruits
Fruit juice	Apple juice

	Orange juice<br>Fruit drink/double drink/ multi fruit drink<br>Other types of fruit juice or fruit drink
Legumes	Legumes
Meat	Boiled liver (on bread)<br>Liver sausage (spread)/pâté/liver pate (on bread)<br>Smoked meat/fricandeau/roast beef (on bread)<br>Bacon/smoked bacon (on bread)<br>Cervelat sausage/sliced sausage/salami (on bread)<br>Luncheon meat/boiled sausage/eel sausage (on bread)<br>Ham (on bread)<br>Other types of meat (on bread)<br>Beef entrecote/beef bratwurst<br>Bacon rashers/bacon bits<br>Pork bratwurst/slavink<br>Pork chop<br>Various types of pork<br>Chicken with skin<br>Chicken without skin<br>Liver<br>Hamburger<br>Minced beef<br>Chopped half and half<br>Smoked sausage/frankfurters<br>Other types of meats<br>Liver sausage (spread)/pâté (in between meals)<br>Boiled liver (in between meals)<br>Other types of meat (in between meals)
Nuts and seeds	Peanut butter<br>Peanuts/nuts (with a hot meal)<br>Peanuts/nuts (in between meals)
Pasta	White pasta<br>Whole wheat pasta
Potatoes	Potatoes with butter/margarine<br>Potatoes without butter/margarine<br>French fries<br>Baked potatoes/rösti<br>Frozen potato slices/potato croquettes<br>Self-fried potatoes, fried in solid frying fat<br>Self-fried potatoes, fried yourself in liquid frying fat<br>Self-fried potatoes, fried in oil
Probiotics and drinks lowering cholesterol and blood pressure	Products with probiotics<br>Drinks lowering cholesterol<br>Drinks lowering blood pressure
Ready-made products	Chinese/Indian ready meals<br>Fast food meals<br>Other types of ready meals<br>Not prepared yourself (takeaway)
Rice	White rice<br>Whole wheat rice
Savoury snacks	Pizza<br>Crisps/pretzels<br>Spread without fish (on bread)<br>Spread with fish (on toast)<br>Salad<br>Croquette<br>Other types of hot snacks

Soft drinks	Soft drinks/lemonade with sugar<br>Energy drink
Soup	Soup with legumes<br>Soup without legumes
Soy products	Soy-based meat substitutes<br>Soy milk<br>Soy drink<br>Soy dessert
Sweets	Chocolate spread<br>Other types of sweet spread<br>Sugar/syrup in yoghurt etc<br>Sugar in coffee<br>Sugar/honey om tea<br>Candy bars<br>Candy<br>Dark chocolate<br>Milk chocolate<br>White chocolate
Tea	Black tea<br>Green tea<br>Tea with licorice extract<br>Herbal tea<br>Other types of tea
Vegan products other than soy	Valess (meat replacer based on dairy)<br>Quorn (meat replacer with mycoprotein)
Vegetables	Green beans/string beans<br>Peas<br>Carrots<br>Brussels sprouts<br>Broccoli<br>Cauliflower<br>White/pointed/green/savoy cabbage<br>Spinach<br>Leek<br>Cut vegetable mix<br>Endive<br>Beets<br>Other types of cooked/stir-fried vegetables<br>Mushrooms<br>Tomato<br>Onions<br>Paprika<br>Lettuce<br>Cucumber<br>Other types of raw vegetables
Water	Water

* Non-alcoholic beer also comprises beer low in alcohol and therefore we included non-alcoholic beer in this food group.
